# Supplementary figures and images for: The SiaABC threonine phosphorylation pathway controls biofilm formation in response to carbon availability in Pseudomonas aeruginosa
Source: PLoS One. 2020 Nov 6;15(11):e0241019. doi: 10.1371/journal.pone.0241019 (PMC7647112; doi:10.1371/journal.pone.0241019)

Glucose

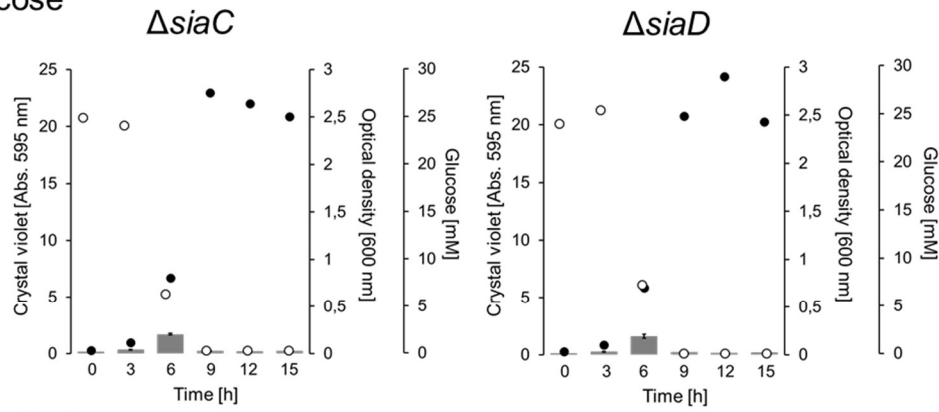

Succinate

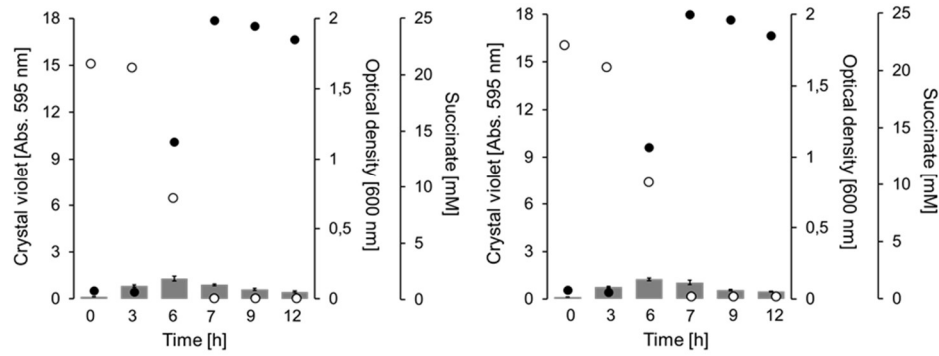

Ethanol

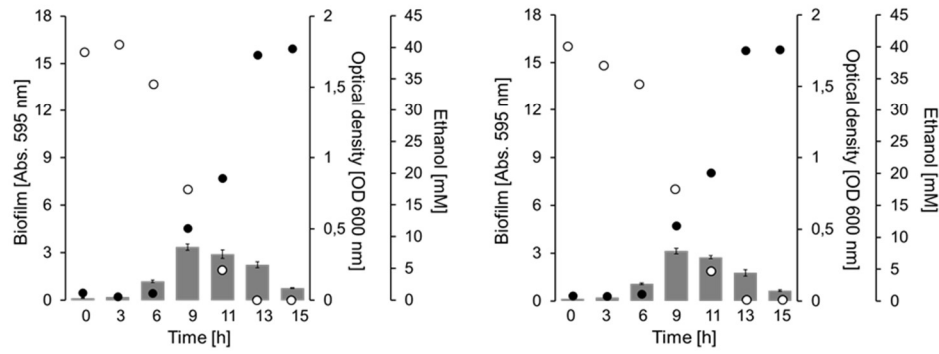

2,3 Butanediol

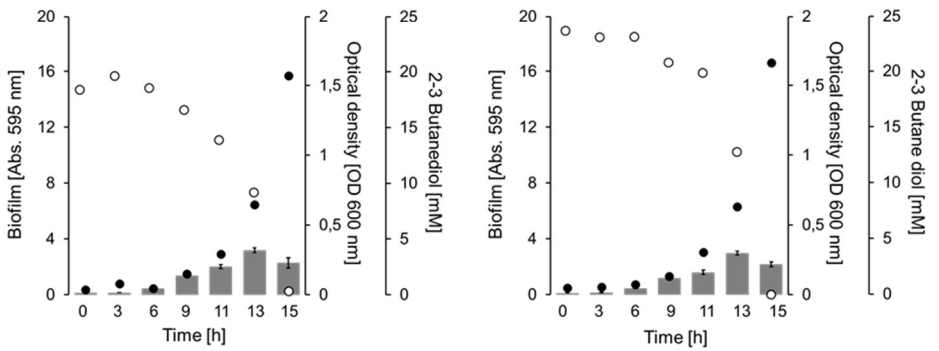

Supplement: S1 Fig — Individual cell-culture treated, 24 well microtiter plates were used to quantify attached biofilms (grey bars), OD600 of supernatants (black dots), and substrate concentrations (open circles) at various time points. Attached biofilms were quantified by a modified crystal violet (CV) staining and error bars represent the standard deviation of four biological replicates (n = 4). OD600 of supernatants and substrate concentrations represent the mean value from the same quadruplicates but quantified from pooled (1:1:1:1 [v/v/v/v]) samples using a photometer, the GO assay kit, and specific HPLC methods (see Material and Method for details). (PDF) [file pone.0241019.s001.pdf]

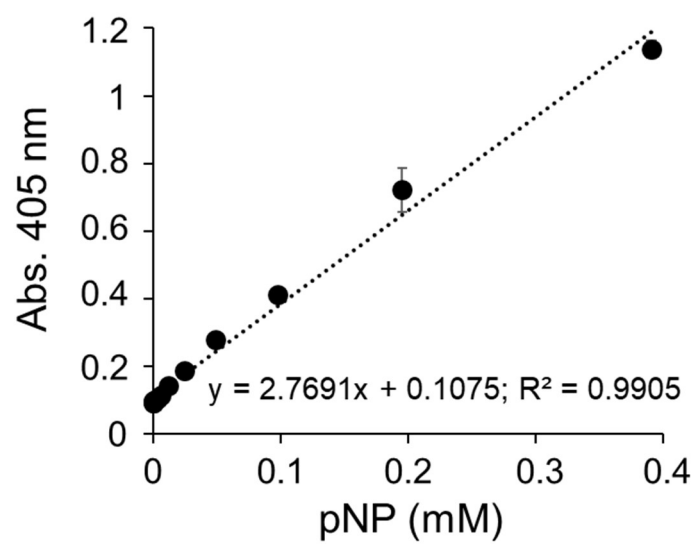

Supplement: S3 Fig — (PDF) [file pone.0241019.s003.pdf]
