# Supplementary material for: The SiaABC threonine phosphorylation pathway controls biofilm formation in response to carbon availability in Pseudomonas aeruginosa
Source: PLoS One. 2020 Nov 6;15(11):e0241019. doi: 10.1371/journal.pone.0241019 (PMC7647112; doi:10.1371/journal.pone.0241019)
Supplement: S2 Fig — A) The purified SiaA phosphatase domain (amino acids 386–663 of the SiaA protein sequence; Genbank ID: NP_248862), the SiaB (Genbank-ID: 248862) and SiaC (Genbank-ID: NP_248860) proteins produced in E. coli after affinity chromatography and subsequent gel filtration. B) The purified SiaC protein (Genbank-ID: NP_248860) produced in the ΔsiaA mutant (SiaCP) after affinity chromatography (eluate) and subsequent protein concentration. (PDF) [file pone.0241019.s002.pdf]

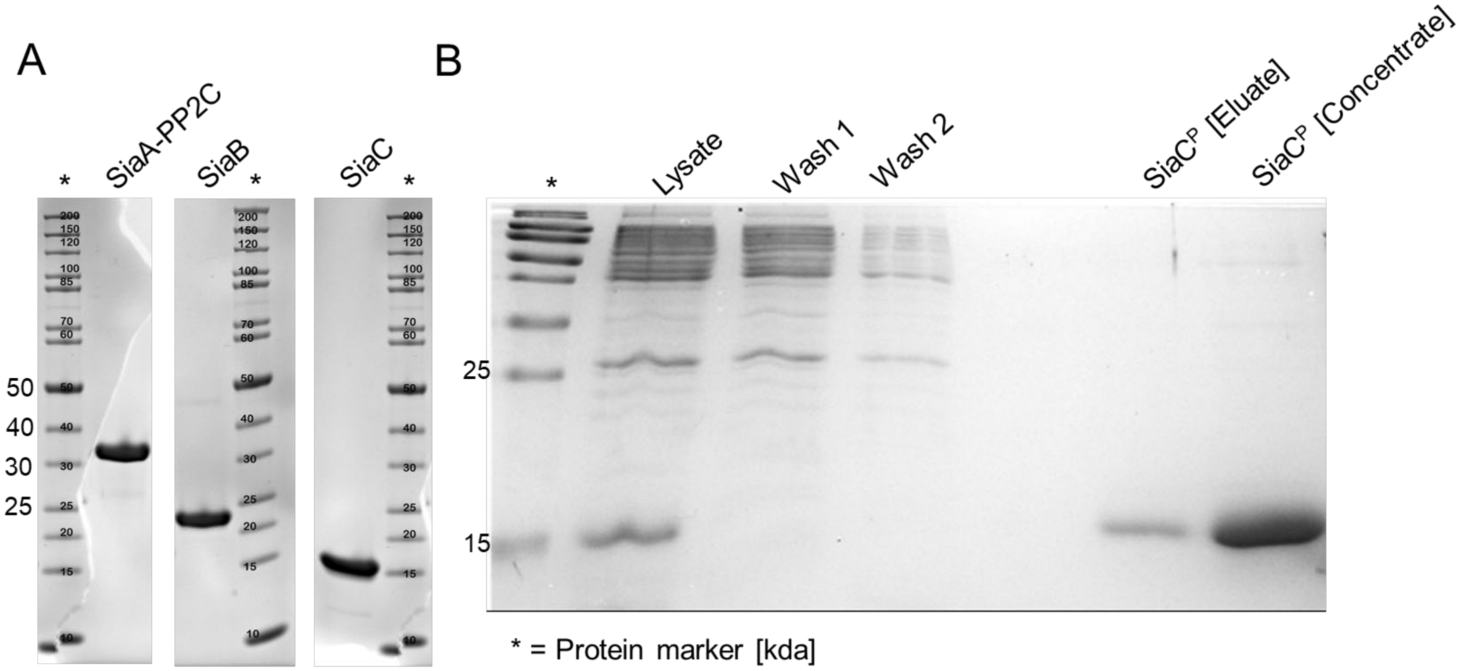

Fig S2: SDS-PAGE analysis of the purified SiaA-PP2C, SiaB, SiaC and SiaAPP2C* protein samples after purification. A) The purified SiaA phosphatase domain (amino acids 386-663 of the SiaA protein sequence; Genbank ID: NP_248862), the SiaB (Genbank-ID: 248862) and SiaC (Genbank-ID: NP_248860) proteins produced in *E. coli* after affinity chromatography and subsequent gel filtration. B) The purified SiaC protein (Genbank-ID: NP_248860) produced in the Δ*siaA* mutant (SiaC^P) after affinity chromatography (eluate) and subsequent protein concentration.
