# Supplementary material for: The SiaABC threonine phosphorylation pathway controls biofilm formation in response to carbon availability in Pseudomonas aeruginosa
Source: PLoS One. 2020 Nov 6;15(11):e0241019. doi: 10.1371/journal.pone.0241019 (PMC7647112; doi:10.1371/journal.pone.0241019)
Supplement: S4 Fig — Conserved residues are highlighted in red. Secondary structure features and residue number of SiaA-PP2C are labelled on top of the alignment. (PDF) [file pone.0241019.s004.pdf]

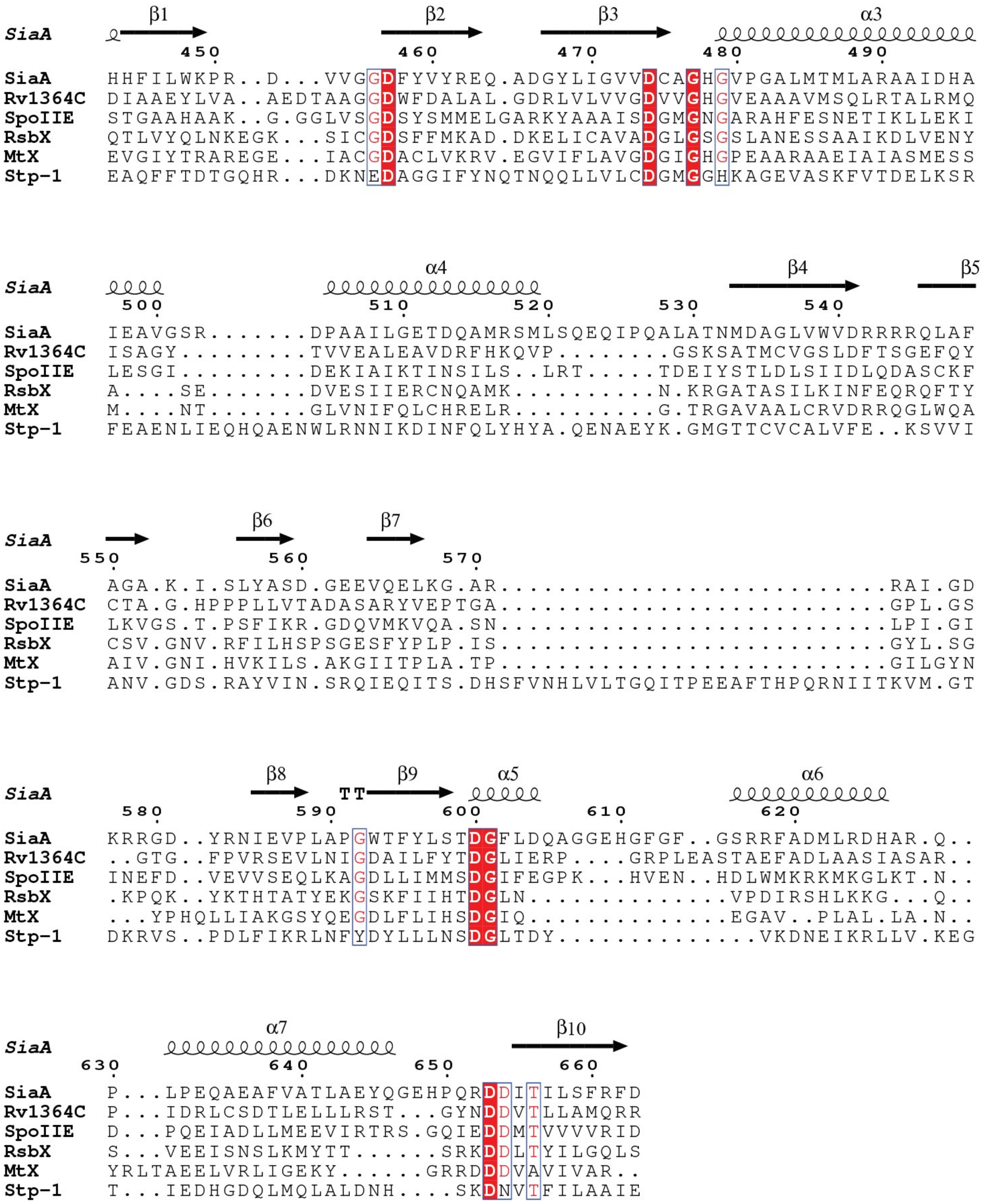
SiaA β1 β2 β3 α3
450 460 470 480 490
SiaA HHFILWKPR..D...VVGGDFYVYREQ.ADGYLIGVVDCAGHGVPGALMTMLARAAIDHA
Rv1364C DIAAEYLVA..AEDTAAGGDWFDALAL.GDRLVLVVGDVVGHGVEAAAVMSQLRTALRMQ
SpoIIE STGAAHAAK..G.GGLVSGDSYSMMELGARKYAAAISDGMGNGARAHFESNETIKLLEKI
RsbX QTLVYQLNKEGK...SICGDSFFMKAD.DKELICAVADGLGSGSLANESSAAIKDLVENY
MtX EVGIYTRAREGE...IACGDACLVKRV.EGVIFLAVGDGIGHGPEAARAAEIAIASMESS
Stp-1 EAQFFTDTGQHR...DKNEDAGGIFYNQTNQQLLVLCDGMGGHKAGEVASKFVTDELKSR
SiaA α4 β4 β5
500 510 520 530 540
SiaA IEAVGSR.......DPAAILGETDQAMRSMLSQEQIPQALATNMDAGLVWVDRRRRQLAF
Rv1364C ISAGY.........TVVEALEAVDRFHKQVP........GSKSATMCVGSLDFTSGEFQY
SpoIIE LESGI.........DEKIAIKTINSILS..LRT.....TDEIYSTLDLSIIDLQDASCKF
RsbX A....SE.......DVESIIERCNQAMK..........N.KRGATASILKINFEQRQFTY
MtX M....NT.......GLVNIFQLCHRELR..........G.TRGAVAALCRVDRRQGLWQA
Stp-1 FEAENLIEQHQAENWLRNNIKDINFQLYHYA.QENAEYK.GMGTTCVCALVFE..KSVVI
SiaA β6 β7
550 560 570
SiaA AGA.K.I.SLYASD.GEEVQELKG.AR.........................RAI.GD
Rv1364C CTA.G.HPPPLLVTADASARYVEPTGA.........................GPL.GS
SpoIIE LKVGS.T.PSFIKR.GDQVMKVQA.SN.........................LPI.GI
RsbX CSV.GNV.RFILHSPSGESFYPLP.IS.........................GYL.SG
MtX AIV.GNI.HVKILS.AKGIITPLA.TP.........................GILGYN
Stp-1 ANV.GDS.RAYVIN.SRQIEQITS.DHSFVNHLVLTGQITPEEAFTHPQRNIITKVM.GT
SiaA β8 TT β9 α5 α6
580 590 600 610 620
SiaA KRRGD..YRNIEVPLAPGWTFYLSTDGFLDQAGGEHGFGF..GSRRFADMLRDHAR.Q..
Rv1364C ..GTG..FPVRSEVLNIGDAILFYTDGLIERP....GRPLEASTAEFADLAASIASAR..
SpoIIE INEFD..VEVVSEQLKAGDLLIMMSDGIFEGPK...HVEN..HDLWMKRKMKGLKT.N..
RsbX .KPQK..YKTHTATYEKGSKFIIHTDGLN.............VPDIRSHLKKG...Q..
MtX ...YPHQLLIAKGSYQEGDLFLIHSDGIQ.............EGAV..PLAL.LA.N..
Stp-1 DKRVS..PDLFIKRLNFYDYLLLNSDGLTDY.............VKDNEIKRLLV.KEG
SiaA α7 β10
630 640 650 660
SiaA P...LPEQAEAFVATLAEYQGEHPQRDDITILSFRFD
Rv1364C P...IDRLCSDTLELLLRST...GYNDDVTLLAMQRR
SpoIIE D...PQEIADLLMEEVIRTRS.GQIEDDMTVVVVRID
RsbX S...VEEISNSLKMYTT......SRKDDLTYILGQLS
MtX YRLTAEELVRLIGEKY......GRRDDDVAVIVAR..
Stp-1 T...IEDHGDQLMQLALDNH....SKDNVTFILAAIE
