# Supplementary material for: The SiaABC threonine phosphorylation pathway controls biofilm formation in response to carbon availability in Pseudomonas aeruginosa
Source: PLoS One. 2020 Nov 6;15(11):e0241019. doi: 10.1371/journal.pone.0241019 (PMC7647112; doi:10.1371/journal.pone.0241019)
Supplement: S1 Table — Analysis of SiaCP (purified from P. aeruginosa) in the absence (A) and presence of the phosphatase domain of SiaA (SiaA-PP2C) (B) after incubation for 2 h at 30°C in phosphatase buffer. SiaC (purified from E. coli) and SiaB incubated in the absence (C) and presence (D) of ATP after incubation for 2 h at 30°C in kinase buffer. Subsequently, the SiaC protein was separated by SDS-PAGE; the SiaC-protein band was cut out of the gel and analysed by PF-MS. (PDF) [file pone.0241019.s005.pdf]

A

Description	Score	Coverage	# Unique Peptides	# PSMs	Area	# AAs	MW [kDa]	calc. pI
SiaC [Pseudomonas aeruginosa PAO1: NC_002516]	5852,44	73,02	8	318	1,3E+09	126	14,5	4,68

Sequence	# PSMs	Modifications	Area	PEP	IonScore	Exp Value	Charge	MH+ [Da]	ΔM [ppm]	RT [min]
AmmDILDLLEEAHQGGRPVSLR	1	M2(Oxidation); M3(Oxidation)	1,7E+06	1,6E-01	27	1,0E-02	4	2483,24	0,95	34,21
AmMDILDLLEEAHQGGRPVSLR	2	M2(Oxidation)	2,0E+06	1,1E-01	11	3,5E-01	4	2467,24	0,19	37,76
AMMDILDLLEEAHQGGRPVSLR	3		9,7E+05	4,8E-03	48	5,4E-05	3	2451,25	1,95	41,34
EDcSFPFAIQAHDE	11	C3(Carbamidomethyl)	3,4E+07	2,7E-02	86	3,6E-09	2	1665,69	1,29	31,67
FLADGQRPLELDLR	75		1,6E+09	1,6E-02	58	3,6E-06	3	1642,89	0,81	27,14
**LLYLNtSSIK**	**34**	**T6(Phospho)**	**1,8E+08**	7,7E-02	44	1,1E-04	**2**	**1231,63**	**0,64**	**31,72**
**LLYLNTSSIK**	**95**		**1,9E+09**	1,2E-02	64	4,0E-07	**2**	**1151,67**	**0,28**	**26,31**
mSDLHIPGTQSTPAIQGDWQAGR	26	M1(Oxidation)	6,9E+07	3,7E-04	74	1,8E-07	3	2482,18	1,18	25,89
MSDLHIPGTQSTPAIQGDWQAGR	22		4,3E+08	2,2E-03	70	5,0E-07	3	2466,19	2,35	27,86
SDLHIPGTQSTPAIQGDWQAGR	6		6,6E+06	4,0E-02	53	2,0E-05	3	2335,14	0,58	27,02
VAELAEEFR	29		2,0E+08	1,5E-02	83	9,0E-09	2	1063,54	0,58	20,16
VAELAEEFREDcSFPFAIQAHDE	14	C12(Carbamidomethyl)	6,3E+07	3,2E-04	92	2,3E-09	3	2710,21	1,12	37,01

B

Description	Score	Coverage	# Unique Peptides	# PSMs	Area	# AAs	MW [kDa]	calc. pI
SiaC [Pseudomonas aeruginosa PAO1: NC_002516]	7095,27	76,98	9	427	3,110E9	126	14,5	4,68

Sequence	# PSMs	Modifications	Area	PEP	IonScore	Exp Value	Charge	MH+ [Da]	ΔM [ppm]	RT [min]
AmmDILDLLEEAHQGGRPVSLR	3	M2(Oxidation); M3(Oxidation)	5,6E+06	5,1E-02	26	1,3E-02	4	2483,24	-0,13	34,40
AmMDILDLLEEAHQGGRPVSLR	12	M2(Oxidation)	7,5E+06	4,7E-03	39	6,1E-04	4	2467,24	-0,01	37,47
AMMDILDLLEEAHQGGRPVSLR	2		3,9E+06	2,2E-02	50	3,6E-05	3	2451,25	1,21	40,80
EDcSFPFAIQAHDE	3	C3(Carbamidomethyl)	9,1E+06	1,9E-02	80	1,3E-08	2	1665,69	1,51	31,76
FLADGQRPLELDLR	121		3,8E+09	9,0E-03	49	2,6E-05	3	1642,89	0,81	28,62
**LLYLNtSSIK**	**7**	**T6(Phospho)**	**1,6E+07**	8,0E-02	**31**	**2,0E-03**	**2**	**1231,63**	**-0,84**	**30,93**
**LLYLNTSSIK**	**178**		**3,7E+09**	7,3E-03	**56**	**2,5E-06**	**2**	**1151,67**	**0,49**	**26,13**
mSDLHIPGTQSTPAIQGDWQAGR	37	M1(Oxidation)	3,0E+08	1,3E-03	73	2,4E-07	3	2482,18	2,29	26,48
MSDLHIPGTQSTPAIQGDWQAGR	41		1,8E+09	9,9E-04	74	2,0E-07	3	2466,18	0,12	28,11
SDLHIPGTQSTPAIQGDWQAGR	5		1,7E+07	2,3E-03	55	1,3E-05	3	2335,15	1,05	26,00
VAELAEEFR	4		5,7E+07	5,3E-03	79	1,8E-08	2	1063,54	0,12	20,73
VAELAEEFREDcSFPFAIQAHDE	12	C12(Carbamidomethyl)	5,8E+07	4,9E-03	85	1,3E-08	3	2710,21	1,12	37,76
WHYDR	2		1,6E+06	1,4E-01	21	7,5E-03	2	776,35	-0,08	7,76

C

Description	Score	Coverage	# Unique Peptides	# PSMs	Area	# AAs	MW [kDa]	calc. pI
SiaC [Pseudomonas aeruginosa PAO1: NC_002516]	9385,72	92,86	9	501	2,115E9	126	14,5	4,68

Sequence	# PSMs	Modifications	Area	PEP	IonScore	Exp Value	Charge	MH+ [Da]	ΔM [ppm]	RT [min]
AmmDILDLLEEAHQGGRPVSLR	34	M2(Oxidation); M3(Oxidation)	2,3E+08	7,6E-12	63	2,6E-06	4	2483,24	0,56	36,38
AmMDILDLLEEAHQGGRPVSLR	60	M2(Oxidation)	3,6E+08	5,3E-11	74	2,1E-07	3	2467,25	1,45	37,62
AMMDILDLLEEAHQGGRPVSLR	9		6,2E+07	6,8E-11	78	5,3E-08	3	2451,25	1,58	41,24
EDcSFPFAIQAHDE	15	C3(Carbamidomethyl)	3,6E+07	4,7E-08	74	4,5E-08	2	1665,69	1,65	31,93
FLADGQRPLELDLR	105		2,5E+09	4,6E-06	50	2,0E-05	3	1642,89	1,03	28,38
**LLYLNtSSIK**	**4**	**T6(Phospho)**	**1,3E+06**	**3,7E-02**	**31**	**1,8E-03**	**2**	**1231,63**	**0,74**	**31,34**
**LLYLNTSSIK**	**135**		**2,9E+09**	**1,4E-06**	**70**	**9,8E-08**	**2**	**1151,67**	**0,71**	**25,31**
LSmQGDSYPENSYELFGQVIDWVER	1	M3(Oxidation)	4,3E+05	3,8E-03	17	1,2E-01	3	2978,36	3,08	51,39
mSDLHIPGTQSTPAIQGDWQAGR	38	M1(Oxidation)	1,6E+08	7,5E-13	80	5,3E-08	3	2482,18	1,99	26,96
MSDLHIPGTQSTPAIQGDWQAGR	32		8,4E+08	1,7E-11	65	1,9E-06	3	2466,19	1,46	29,36
SDLHIPGTQSTPAIQGDWQAGR	7		9,5E+06	5,5E-10	58	6,8E-06	3	2335,14	-0,20	27,47
VAELAEEFR	2		1,6E+07	7,1E-09	66	4,4E-07	2	1063,54	0,35	21,04
VAELAEEFREDcSFPFAIQAHDE	59	C12(Carbamidomethyl)	9,9E+08	1,0E-14	94	1,6E-09	3	2710,21	1,66	38,88

D

Description	Score	Coverage	# Unique Peptides	# PSMs	Area	# AAs	MW [kDa]	calc. pI
SiaC [Pseudomonas aeruginosa PAO1: NC_002516]	7632,28	76,98	9	428	1,494E9	126	14,5	4,68

Sequence	# PSMs	Modifications	Area	PEP	IonScore	Exp Value	Charge	MH+ [Da]	ΔM [ppm]	RT [min]
AmmDILDLLEEAHQGGRPVSLR	14	M2(Oxidation); M3(Oxidation)	6,0E+07	3,1E-08	54	1,7E-05	3	2483,24	1,40	33,95
AmMDILDLLEEAHQGGRPVSLR	32	M2(Oxidation)	5,8E+07	3,1E-09	79	5,7E-08	3	2467,26	4,87	37,37
AMMDILDLLEEAHQGGRPVSLR	2		7,6E+06	4,7E-09	51	2,7E-05	3	2451,25	0,61	40,99
EDcSFPFAIQAHDE	19	C3(Carbamidomethyl)	9,7E+07	1,1E-09	76	3,6E-08	2	1665,69	0,48	31,85
FLADGQRPLELDLR	83		1,8E+09	1,0E-08	54	9,3E-06	3	1642,89	0,03	29,62
**LLYLNtSSIK**	**91**	**T6(Phospho)**	**1,5E+09**	**3,2E-05**	**60**	**2,7E-06**	**2**	**1231,63**	**1,24**	**39,61**
**LLYLNTSSIK**	**30**		**2,7E+08**	**8,9E-09**	**66**	**2,3E-07**	**2**	**1151,67**	**0,49**	**25,10**
mSDLHIPGTQSTPAIQGDWQAGR	48	M1(Oxidation)	4,2E+08	2,2E-15	100	4,3E-10	3	2482,18	2,29	26,70
MSDLHIPGTQSTPAIQGDWQAGR	43		1,1E+09	1,9E-12	75	2,0E-07	3	2466,19	2,27	28,50
SDLHIPGTQSTPAIQGDWQAGR	5		1,1E+07	1,2E-08	53	1,9E-05	3	2335,15	2,39	26,68
VAELAEEFR	5		4,6E+07	4,1E-09	79	1,8E-08	2	1063,54	0,24	21,09
VAELAEEFREDcSFPFAIQAHDE	54	C12(Carbamidomethyl)	7,6E+08	5,0E-14	88	7,1E-09	3	2710,22	2,40	37,59
WHYDR	2		1,4E+06	2,0E-01	21	7,8E-03	2	776,35	0,08	7,76
