# Supplementary material for: The SiaABC threonine phosphorylation pathway controls biofilm formation in response to carbon availability in Pseudomonas aeruginosa
Source: PLoS One. 2020 Nov 6;15(11):e0241019. doi: 10.1371/journal.pone.0241019 (PMC7647112; doi:10.1371/journal.pone.0241019)
Supplement: S2 Table — (PDF) [file pone.0241019.s006.pdf]

	SiaA Native	SiaA SeMet	SiaC Native	SiaC SeMet
**Wavelength (Å)**	0.9764	0.979	0.9801	0.9766
**Resolution range (Å)**	49.81 - 2.094 (2.169 - 2.094)	27.35 - 2.49 (2.579 - 2.49)	57.96 - 1.735 (1.797 - 1.735)	38.74 - 2.94 (3.045 - 2.94)
**Space group**	P 21 21 21	P 21 21 21	I 2 2 2	P 31 2 1
**Unit cell (Å)**	51.17 110.48 111.6 90 90 90	51.354 109.416 111.46 90 90 90	35.4 72.8 95.8 90 90 90	77.482 77.482 85.02 90 90 120
**Total reflections**	205960 (19009)	606993 (59041)	116718 (7271)	130598 (12091)
**Unique reflections**	37739 (3471)	22684 (2229)	13113 (1176)	6550 (603)
**Multiplicity**	5.5 (5.5)	26.8 (26.5)	8.9 (6.2)	19.9 (19.9)
**Completeness (%)**	99.18 (93.11)	99.85 (99.82)	98.94 (90.81)	99.53 (96.20)
**Mean I/sigma (I)**	7.20 (0.99)	20.17 (1.65)	12.05 (0.90)	21.22 (1.56)
**Wilson B-factor (Å^2)**	41.65	66.72	34.21	97.73
**R-merge**	0.1603 (1.432)	0.1411 (1.813)	0.09846 (1.129)	0.1306 (1.444)
**CC1/2**	0.993 (0.461)	0.998 (0.752)	0.998 (0.62)	0.999 (0.784)
**R-work**	0.2177 (0.3346)	0.2055 (0.2768)	0.1972 (0.3956)	0.2122 (0.3450)
**R-free**	0.2522 (0.3782)	0.2542 (0.3665)	0.2348 (0.4305)	0.2812 (0.4126)
**RMS (bonds) (Å)**	0.014	0.014	0.008	0.015
**RMS (angles) (°)**	1.71	1.77	1.19	1.82
**Ramachandran outliers (%)**	0.00	0.00	0.00	0.41
**Rotamer outliers (%)**	3.73	4.07	0.00	12.50
**Clashscore**	2.80	3.23	2.65	8.62
**Average B-factor (Å^2)**	53.79	71.23	43.58	106.10
