# Supplementary material for: The SiaABC threonine phosphorylation pathway controls biofilm formation in response to carbon availability in Pseudomonas aeruginosa
Source: PLoS One. 2020 Nov 6;15(11):e0241019. doi: 10.1371/journal.pone.0241019 (PMC7647112; doi:10.1371/journal.pone.0241019)
Supplement: S3 Table — (PDF) [file pone.0241019.s007.pdf]

Strains	Relevant features	Reference
P. aeruginosa		
PAO1	Wild-type *Pseudomonas aeruginosa*	Holloway Collection
Δ*siaA*	PAO1 with a 6 bp deletion in *siaA*	[1]
Δ*siaB*	PAO1 with a markerless *loxP*-site insertion in *siaB* (PA0171)	This study
Δ*siaC*	PAO1 with a markerless *loxP*-site insertion in *siaC* (PA0170)	This study
Δ*siaD*	PAO1 with a markerless *res*-site insertion in *siaD* (KO0169)	[1]
PW1292	MPAO1 with transposon phoAwp091G03 inserted at bp 311 of the coding region of the *siaB* gene[	[2]
PW1290	MPAO1 with transposon phoAwp09q2A10 inserted at bp 181 of the coding region of the *siaC* gene	[2]
E. coli		
DH5α	*fhuA2 lac(del)U169 phoA glnV44 Φ80' lacZ(del)M15 gyrA96 recA1 relA1 endA1 thi-1 hsdR17*	[3]
S17-λpir	Tp^r Sm^r *recA, thi, pro, hsdR*-M^+RP4: 2-Tc:Mu: Km^r Tn7 λpir	[4]
BL21(DE3) Rosetta T1R	*E. coli* str. B F^- *ompT gal dcm lon* $hsdS_B(r_B^- m_B^-)$ λ(DE3 [*lacI lacUV5-T7p07 ind1 sam7 nin5*]) $[malB^+]_{K-12}(\lambda^S)$ *tonA*	NTU Protein Production Plattform
**Plasmids**		
pCR2.1	TOPO TA cloning plasmid, Amp^r, Kan^r	Invitrogen
pBBR	Broad host range expression plasmid pBBR1MCS-5, Gm^r	[5]
pJEM1	Rhamnose inducible broad host range expression plasmid, Kan^r	[6]
pNIC28-BSA4	Protein production plasmid, N-terminal His6, TEV-cleavable MHHHHHHSSGVDLGTENLYFQ*SM, Kan^r	[7]
pNIC-CTHF	Protein production plasmid, Cleavable C-terminal His6-FLAG tag, Kan^r	[7]
pNIC[SiaA]	pNIC28-BSA4 harbouring the partial *siaA* gene (C-terminal phosphatase domain from amino acid 386-663) for the production of a N-terminal His6-TEV-SiaA allele (SiaA-PP2C).	This study
pNIC[SiaB]	pNIC28-BSA4 for the production of a N-terminal His6-TEC SiaB allele	This study
pNIC[SiaC]	pNIC28-BSA4 for the production of a N-terminal His6-TEV-SiaC allele	This study
pJEM[SiaC]	pJEM1 harbouring the *siaC* gene for the production of a N-terminal His6-TEV-SiaC allele in *Pseudomonas aeruginosa* strains	This study
pCre1	Plasmid for the expression of Cre recombinase	[8]
**Phage**		
E79tv2	Generalised transducing phage	[9]

References

1. Klebensberger, J., Birkenmaier, A., Geffers, R., Kjelleberg, S. & Philipp, B. SiaA and SiaD are essential for inducing autoaggregation as a specific response to detergent stress in *Pseudomonas aeruginosa.* Environ Microbiol. 2009;11: 3073–3086.
2. Jacobs, M. A. *et al.* Comprehensive transposon mutant library of *Pseudomonas aeruginosa*. Proc Natl Acad Sci. 2003;100: 14339–14344.
3. Bethesda Research Laboratories. *E. coli* DH5 alpha competent cells. Focus-Bethesda Res Lab. 1986;8:9.
4. Simon, R., Priefer, U. & Pühler, A. A broad host range mobilization system for in vivo genetic engineering: Transposon mutagenesis in gram negative bacteria. *Bio/Technology* **1,** 784–791 (1983).Lämmli, U. K. Cleavage of Structural Proteins during the Assembly of the Head of Bacteriophage T4. Nature**.** 1970;227: 680–685.
5. Kovach, M. E. *et al.* Four new derivatives of the broad-host-range cloning vector pBBR1MCS, carrying different antibiotic-resistance cassettes. Gene. 1995;166: 175-176.
6. Jeske, M. & Altenbuchner, J. The *Escherichia coli* rhamnose promoter rhaP BAD is in *Pseudomonas putida* KT2440 independent of Crp–cAMP activation. Appl Microbiol Biotechnol. 2010;85: 1923–1933.
7. Savitsky, P. *et al.* High-throughput production of human proteins for crystallization: The SGC experience. J Struct Biol. 2010;172: 3–13.
8. Bailey, J. A. *et al.* Recent segmental duplications in the human genome. Science. 2002;297: 1003–1007.
9. Morgan, A. F. Transduction of *Pseudomonas aeruginosa* with a mutant of bacteriophage E79. J Bacteriol. 1979;139: 137–140.
