# Supplementary material for: The SiaABC threonine phosphorylation pathway controls biofilm formation in response to carbon availability in Pseudomonas aeruginosa
Source: PLoS One. 2020 Nov 6;15(11):e0241019. doi: 10.1371/journal.pone.0241019 (PMC7647112; doi:10.1371/journal.pone.0241019)
Supplement: S4 Table — (PDF) [file pone.0241019.s008.pdf]

Gene/Construct	Primer name and sequence (5' to 3')	
SiaA-PP2C	SiaA-f6117	TACTTCCAATCCATGCGGCACACCGCCGAGCT
	SiaA-r6103	TATCCACCTTTACTGTCAGTCGAATCGGAAGGACAGG
SiaB	SiaB-f6118	TACTTCCAATCCATGATGGAAACGCTAGACCTGCT
	SiaB-r6104	TATCCACCTTTACTGTCATCAGATCACGGCGCGCAG
SiaC	SiaC-f6119	TACTTCCAATCCATGATGAGTGACCTGCACATACC
	SiaC-r6105	TATCCACCTTTACTGTCACTACTCGTCGTGGGCCTG
His6-TEV-SiaC	His_TEV_SiaC_F	ACAATTCTTAAGAAGGAGATATACAATGCACCATCATCATCATCATTCTTC
	His_TEV_SiaC_R	GCTTCCGGTAGTCAATAAACCGGTACTACTCGTCGTGGGCCT
