# Supplementary material for: The SiaABC threonine phosphorylation pathway controls biofilm formation in response to carbon availability in Pseudomonas aeruginosa
Source: PLoS One. 2020 Nov 6;15(11):e0241019. doi: 10.1371/journal.pone.0241019 (PMC7647112; doi:10.1371/journal.pone.0241019)
Supplement: S1 File — (PDF) [file pone.0241019.s009.pdf]

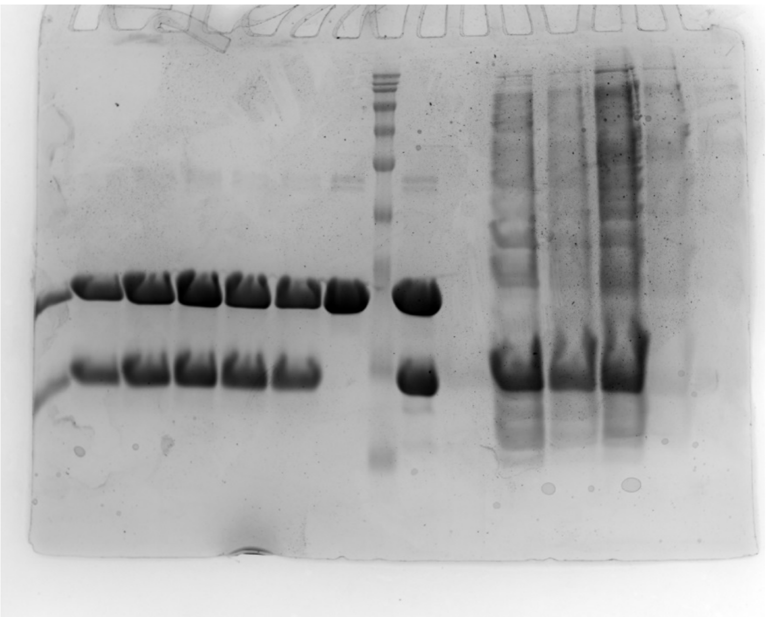

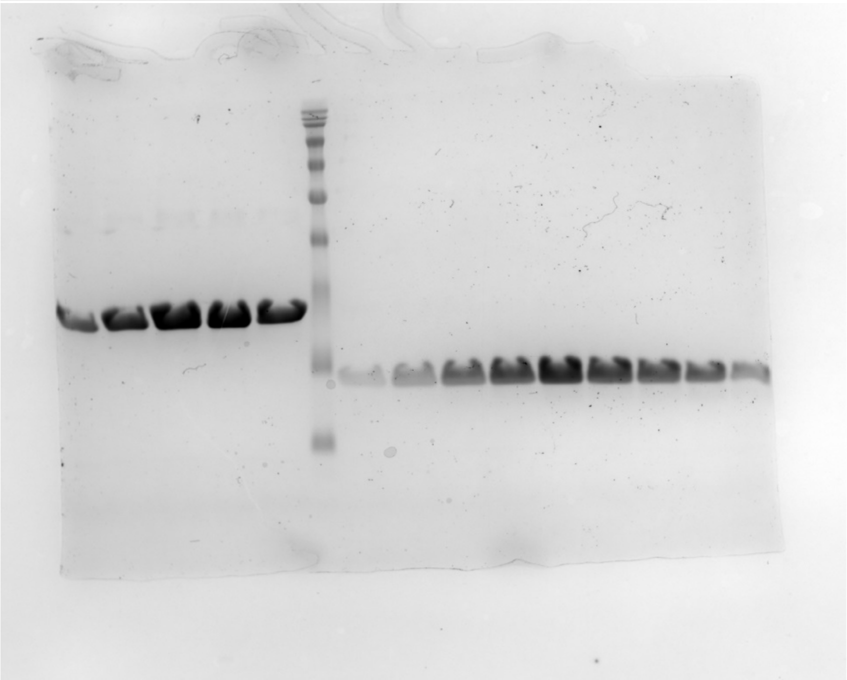

Original gel pictures for Figure 6E (SiaB/SiaC complex).

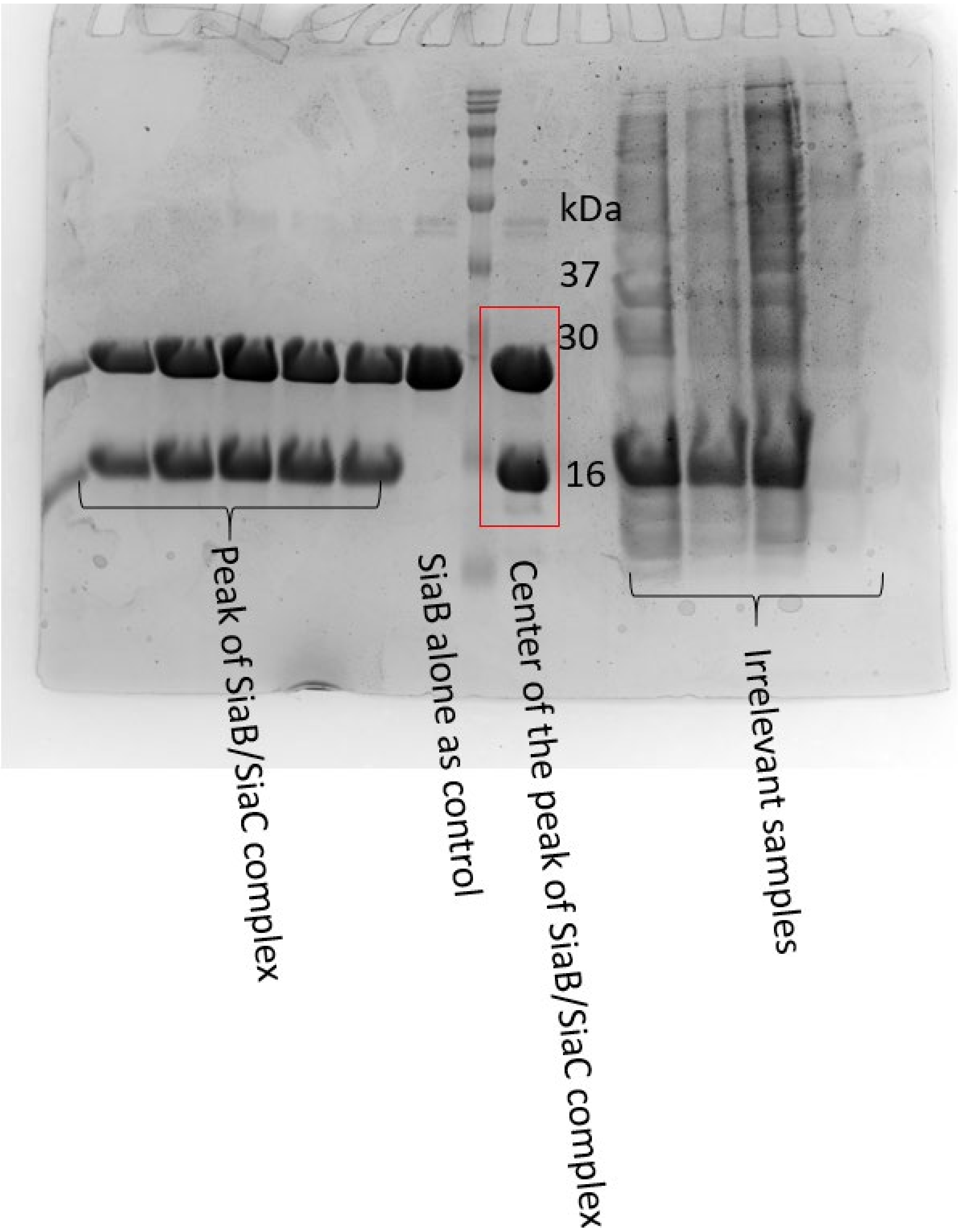

Original gel pictures for Figure 6E (SiaB/SiaC complex) with annotation. The area in red rectangle was cropped out for publication.

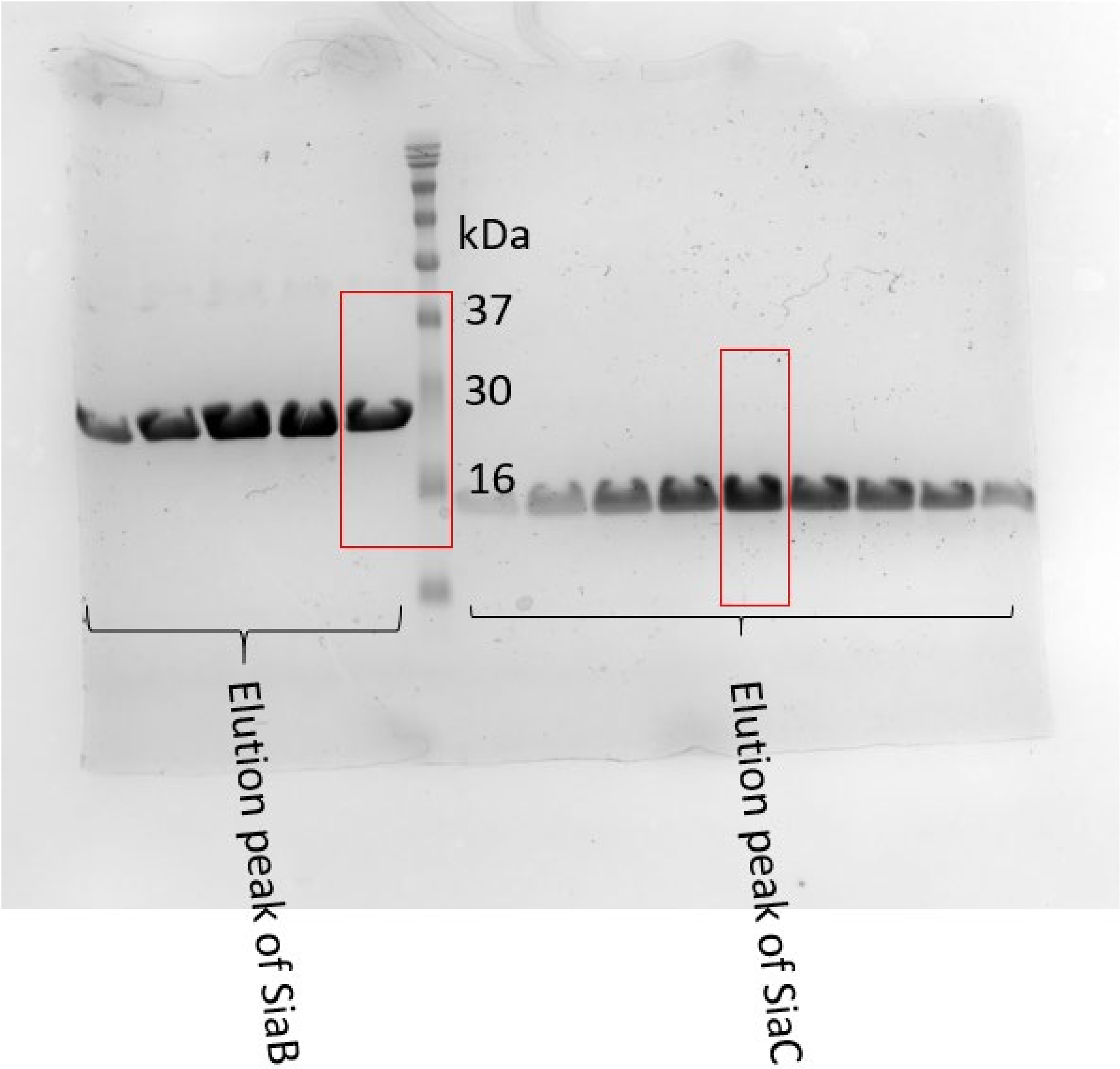

Original gel picture for Figure 6E (SiaB/SiaC complex dissociation) with annotation. The area in red rectangle was cropped out for publication.

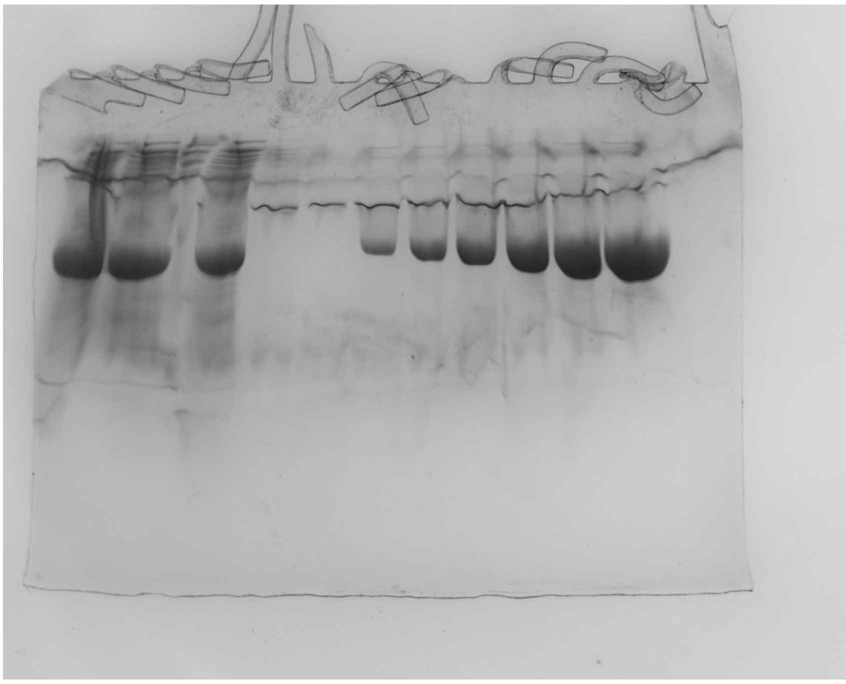

Original gel picture for Figure 6E (SiaB/SiaC complex dissociation) with annotation. The area in red rectangle was cropped out for publication.

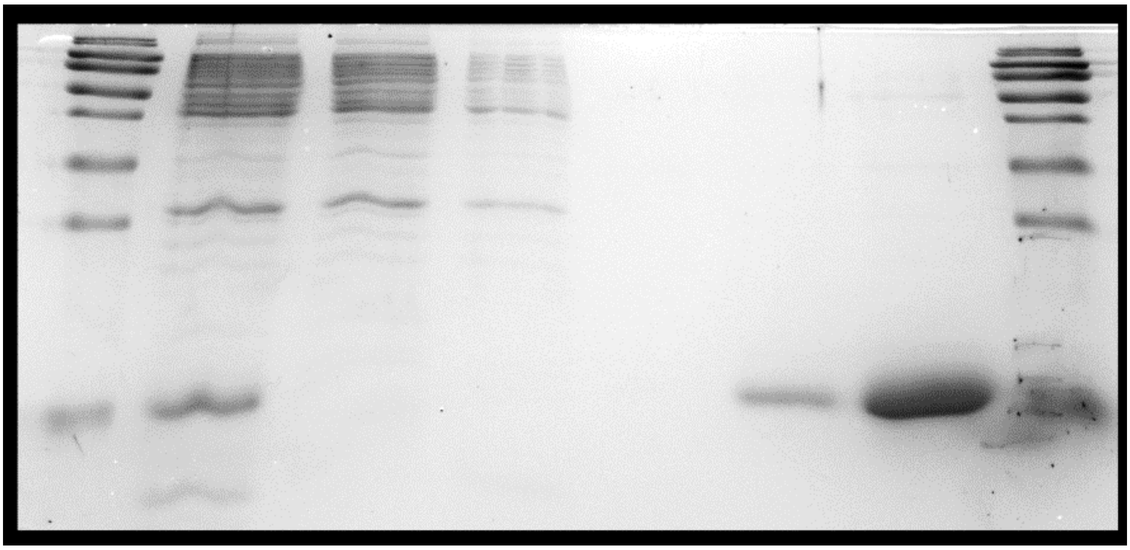

Original gel picture for Figure S2B (purification of SiaC^{P})
